# 793. Rifabutin Use in Staphylococcal Prosthetic Material Infections: A Case Series

**DOI:** 10.1093/ofid/ofab466.989

**Published:** 2021-12-04

**Authors:** Miranda Monk, Ramy H Elshaboury, Sandra B Nelson, Monique R Bidell

**Affiliations:** Massachusetts General Hospital, Medford, Massachusetts

## Abstract

**Background:**

Bacterial biofilm formation is of clinical concern among patients with staphylococcal infections involving prosthetic material. While rifampin has in-vitro, animal and clinical data to support its adjunctive role in these types of infections, its potent induction of multiple cytochrome P450 enzymes and P-glycoprotein transport system proteins can pose significant drug-drug interactions. Rifabutin has comparable in-vitro anti-staphylococcal activity but less drug-drug interaction potential than rifampin. However, minimal clinical data exists to support rifabutin use as adjunctive treatment of prosthetic infections.

**Methods:**

This case series describes 7 patients who received adjunctive rifabutin for staphylococcal prosthetic material infections between February 2018 and January 2021 at Massachusetts General Hospital. The primary outcome of infection recurrence was defined as need for surgical intervention for suspected or proven recurrence infection within 6 months after starting rifabutin therapy. Incidence of adverse effects was the main secondary outcome.

**Results:**

Most patients (6/7) had methicillin-sensitive *S. aureus* (MSSA) and one patient had *S. epidermidis* infection. Three patients had spinal fusion hardware infection, one patient had hardware-associated spinal osteomyelitis/ diskitis with epidural abscess, two patients had prosthetic joint infection, and one patient had MSSA bacteremia with left ventricular assistance device involvement. All patients except one underwent surgical management prior to starting rifabutin. Infection recurrence was noted in one of seven patients who required surgical washout. Adverse events were uncommon (n=1 treatment-related nausea, n=1 leukopenia).

**Conclusion:**

This small case series suggests favorable outcomes with use of rifabutin instead of rifampin for staphylococcal infections with prosthetic material involvement.

**Disclosures:**

**Sandra B. Nelson, MD**, **UpToDate** (Other Financial or Material Support, author)

